# Aqua­(4-carb­oxy­pyridine-2,6-dicarboxyl­ato-κ^3^ *O* ^2^,*N*,*O* ^6^)(1,10-phenanthroline-κ^2^ *N*,*N*′)nickel(II)

**DOI:** 10.1107/S1600536811026055

**Published:** 2011-07-09

**Authors:** Qin Zou, Jian-fei Wang, Jian-li Lin

**Affiliations:** a Center of Applied Solid State Chemistry Research, Ningbo University, Ningbo, Zhejiang 315211, People’s Republic of China

## Abstract

The title compound, [Ni(C_8_H_3_NO_6_)(C_12_H_8_N_2_)(H_2_O)], contains an Ni^II^ ion, a 1,10-phenanthroline (phen) ligand, a 4-carb­oxy­pyridine-2,6-dicarboxyl­ate (Hptc^2−^) anion and a coordinated water mol­ecule. The Ni^II^ atom exhibits a distorted octa­hedral N_3_O_3_ environment. O—H⋯O hydrogen bonding between coordinated water and carboxyl­ate O atoms, as well as π–π stacking inter­actions [inter­planar distances between phen rings = 3.293 (2) Å] lead to a supermolecular assembly.

## Related literature

For the synthesis of pyridine-2,4,6-tricarb­oxy­lic acid, see: Syper *et al.* (1980 ▶). For related structures, see: Ma *et al.* (2002 ▶); Ramadevi *et al.* (2006 ▶); Harrison *et al.* (2006 ▶).
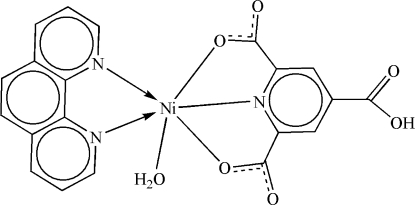


## Experimental

### 

#### Crystal data

[Ni(C_8_H_3_NO_6_)(C_12_H_8_N_2_)(H_2_O)]
*M* *_r_* = 466.04
Monoclinic, 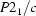

*a* = 6.8387 (14) Å
*b* = 13.421 (3) Å
*c* = 19.676 (4) Å
β = 91.87 (3)°
*V* = 1805.0 (6) Å^3^
*Z* = 4
Mo *K*α radiation
μ = 1.13 mm^−1^
*T* = 293 K
0.24 × 0.22 × 0.10 mm

#### Data collection

Rigaku R-AXIS RAPID diffractometer
Absorption correction: multi-scan (*ABSCOR*; Higashi, 1995 ▶) *T*_min_ = 0.763, *T*_max_ = 0.893
17250 measured reflections
4058 independent reflections
2573 reflections with *I* > 2σ(*I*)
*R*_int_ = 0.054

#### Refinement

*R*[*F* ^2^ > 2σ(*F* ^2^)] = 0.044
*wR*(*F* ^2^) = 0.142
*S* = 1.19
4058 reflections
280 parameters
H-atom parameters constrained
Δρ_max_ = 1.34 e Å^−3^
Δρ_min_ = −1.52 e Å^−3^

### 

Data collection: *RAPID-AUTO* (Rigaku, 1998 ▶); cell refinement: *RAPID-AUTO*; data reduction: *CrystalStructure* (Rigaku/MSC, 2004 ▶); program(s) used to solve structure: *SHELXS97* (Sheldrick, 2008 ▶); program(s) used to refine structure: *SHELXL97* (Sheldrick, 2008 ▶); molecular graphics: *SHELXTL* (Sheldrick, 2008 ▶); software used to prepare material for publication: *SHELXL97*.

## Supplementary Material

Crystal structure: contains datablock(s) global, I. DOI: 10.1107/S1600536811026055/pv2419sup1.cif

Structure factors: contains datablock(s) I. DOI: 10.1107/S1600536811026055/pv2419Isup2.hkl

Additional supplementary materials:  crystallographic information; 3D view; checkCIF report

## Figures and Tables

**Table 1:** Hydrogen-bond geometry (Å, °)

*D*—H⋯*A*	*D*—H	H⋯*A*	*D*⋯*A*	*D*—H⋯*A*
O3—H3*A*⋯O2^i^	0.85	1.70	2.550 (5)	178
O7—H7*A*⋯O5^ii^	0.85	1.87	2.702 (5)	167
O7—H7*B*⋯O5^iii^	0.85	2.00	2.783 (5)	152

Symmetry codes: (i) 
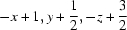
; (ii) 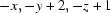; (iii) 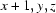.

**Fig. 1.**
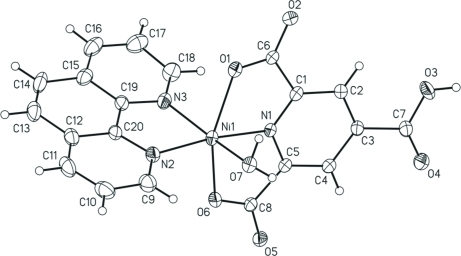
ORTEP view of the title compound. The dispalcement ellipsoids are drawn at 35% probability level.

**Fig. 2.**
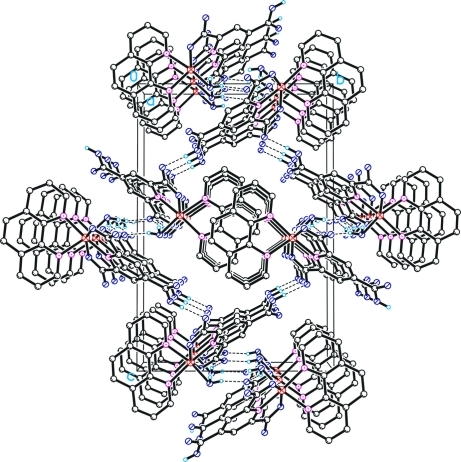
Packing diagram of the title crystal structure viewed along [100]. O—H···O hydrogen bonds are shown as dashed line.

## supplementary crystallographic information

### Comment

The asymmetric unit of the title compound contains a Ni^II^ ion, a 1,10-phenanthroline ligand, a 4-carboxypyridine-2,6-dicarboxylate (Hptc^2-^) anion and a coordinated molecule of water. The Hptc^2-^ anion adopts a η^3^µ_1_ coordination mode and chelates the Ni^II^ atom through the pyridine N atom and two neighboring carboxylate O atoms. The Ni^II^ atoms is six-coordinated by two N atoms from phen, one nitrogen from Hptc^2-^, two oxygen atoms from Hptc^2-^ and one oxygen from coordinated water, in a octahedral N_3_O_3_ environment. The π–π stacking interactions between the parallel phen rings with the interplanar distance of 3.293 (2)Å interlink the [Ni(Hptc)(phen)(H_2_O)] units to form one-dimensional chains, which further grow into three-dimensional supramolecular construction by hydrogen bonding O—H···O interactions between coordinated water molecules and carboxylate groups.

### Experimental

The ligand (H_3_ptc) was synthesized by oxidization of pyridine-2,4,6-trimethyl with potassium permanganate as reported in the literature (Syper *et al.*, 1980). A solution of Ni(ClO_4_)_2_.6H_2_O (0.0731 g, 0.2 mmol), H_3_ptc (0.0425 g, 0.2 mmol), phen (0.0396 g, 0.2 mmol) in H_2_O (8.0 ml) wasy sealed in a 23 ml Teflon-lined stainless-steel autoclave, which was heated to 413 K and kept at this temperature for 3 days, then the reactor was slow cooled to room temperature at a rate of 5 K/h, green crystals were collected after filtration.

### Refinement

H atoms bonded to C atoms were placed in their geometrically calculated positions and refined using the riding model, with C–H distances 0.93Å and *U*_iso_(H) = 1.2 *U*_eq_(C). H atoms attached to O atoms were found in a difference Fourier map and then refined using the riding model, with O–H distances fixed at 0.85Å and *U*_iso_(H) values set at 1.2 *U*_eq_(O). The final difference map showed residual electron density close to the Ni-atom which was essentially meaningless.

### Crystal data

**Table:**

[Ni(C_8_H_3_NO_6_)(C_12_H_8_N_2_)(H_2_O)]	*F*(000) = 952
*M**_r_* = 466.04	*D*_x_ = 1.715 Mg m^−^^3^
Monoclinic, *P*2_1_/*c*	Mo *K*α radiation, λ = 0.71073 Å
Hall symbol: -P 2ybc	Cell parameters from 10705 reflections
*a* = 6.8387 (14) Å	θ = 3.0–27.4°
*b* = 13.421 (3) Å	µ = 1.13 mm^−^^1^
*c* = 19.676 (4) Å	*T* = 293 K
β = 91.87 (3)°	Chip, green
*V* = 1805.0 (6) Å^3^	0.24 × 0.22 × 0.10 mm
*Z* = 4	

### Data collection

**Table:**

Rigaku R-AXIS RAPID diffractometer	4058 independent reflections
Radiation source: fine-focus sealed tube	2573 reflections with *I* > 2σ(*I*)
graphite	*R*_int_ = 0.054
Detector resolution: 0 pixels mm^-1^	θ_max_ = 27.5°, θ_min_ = 3.0°
ω scan	*h* = −8→7
Absorption correction: multi-scan (*ABSCOR*; Higashi, 1995)	*k* = −17→17
*T*_min_ = 0.763, *T*_max_ = 0.893	*l* = −25→24
17250 measured reflections	

### Refinement

**Table:**

Refinement on *F*^2^	Primary atom site location: structure-invariant direct methods
Least-squares matrix: full	Secondary atom site location: difference Fourier map
*R*[*F*^2^ > 2σ(*F*^2^)] = 0.044	Hydrogen site location: inferred from neighbouring sites
*wR*(*F*^2^) = 0.142	H-atom parameters constrained
*S* = 1.19	*w* = 1/[σ^2^(*F*_o_^2^) + (0.0123*P*)^2^ + 8.1487*P*] where *P* = (*F*_o_^2^ + 2*F*_c_^2^)/3
4058 reflections	(Δ/σ)_max_ < 0.001
280 parameters	Δρ_max_ = 1.34 e Å^−^^3^
0 restraints	Δρ_min_ = −1.52 e Å^−^^3^

### Special details

**Table:**

Geometry. All e.s.d.'s (except the e.s.d. in the dihedral angle between two l.s. planes) are estimated using the full covariance matrix. The cell e.s.d.'s are taken into account individually in the estimation of e.s.d.'s in distances, angles and torsion angles; correlations between e.s.d.'s in cell parameters are only used when they are defined by crystal symmetry. An approximate (isotropic) treatment of cell e.s.d.'s is used for estimating e.s.d.'s involving l.s. planes.
Refinement. Refinement of *F*^2^ against ALL reflections. The weighted *R*-factor *wR* and goodness of fit *S* are based on *F*^2^, conventional *R*-factors *R* are based on *F*, with *F* set to zero for negative *F*^2^. The threshold expression of *F*^2^ > σ(*F*^2^) is used only for calculating *R*-factors(gt) *etc*. and is not relevant to the choice of reflections for refinement. *R*-factors based on *F*^2^ are statistically about twice as large as those based on *F*, and *R*- factors based on ALL data will be even larger.

### Fractional atomic coordinates and isotropic or equivalent isotropic displacement parameters (Å^2^)

**Table:**

	*x*	*y*	*z*	*U*_iso_*/*U*_eq_	
Ni1	0.26648 (9)	0.77711 (4)	0.53877 (3)	0.02863 (18)	
N1	0.2227 (6)	0.8906 (3)	0.60244 (19)	0.0268 (9)	
C1	0.3686 (7)	0.9196 (3)	0.6443 (2)	0.0309 (11)	
C2	0.3585 (7)	1.0087 (3)	0.6793 (2)	0.0304 (11)	
H2A	0.4603	1.0289	0.7086	0.036*	
C3	0.1919 (7)	1.0673 (3)	0.6695 (2)	0.0286 (10)	
C4	0.0397 (7)	1.0350 (3)	0.6258 (2)	0.0297 (10)	
H4A	−0.0730	1.0730	0.6191	0.036*	
C5	0.0617 (7)	0.9450 (3)	0.5930 (2)	0.0260 (10)	
C6	0.5378 (7)	0.8459 (3)	0.6473 (2)	0.0291 (10)	
O1	0.5323 (5)	0.7781 (3)	0.60344 (17)	0.0341 (8)	
O2	0.6618 (5)	0.8555 (3)	0.69549 (19)	0.0418 (9)	
C7	0.1719 (8)	1.1668 (4)	0.7044 (2)	0.0343 (11)	
O3	0.3151 (6)	1.1847 (3)	0.7480 (2)	0.0595 (13)	
H3A	0.3243	1.2410	0.7678	0.071*	
O4	0.0343 (6)	1.2209 (3)	0.6934 (2)	0.0495 (10)	
C8	−0.0789 (7)	0.9010 (3)	0.5395 (2)	0.0275 (10)	
O5	−0.2403 (5)	0.9434 (2)	0.52867 (18)	0.0337 (8)	
O6	−0.0182 (5)	0.8251 (2)	0.50894 (17)	0.0339 (8)	
N2	0.2834 (6)	0.6679 (3)	0.4662 (2)	0.0322 (9)	
C9	0.3157 (8)	0.6781 (4)	0.4005 (3)	0.0422 (13)	
H9A	0.3303	0.7417	0.3828	0.051*	
C10	0.3286 (9)	0.5957 (5)	0.3570 (3)	0.0497 (15)	
H10A	0.3494	0.6053	0.3109	0.060*	
C11	0.3106 (8)	0.5023 (5)	0.3821 (3)	0.0498 (16)	
H11A	0.3230	0.4474	0.3537	0.060*	
C12	0.2735 (7)	0.4888 (4)	0.4508 (3)	0.0404 (13)	
C13	0.2495 (8)	0.3931 (4)	0.4819 (4)	0.0489 (16)	
H13A	0.2571	0.3358	0.4556	0.059*	
C14	0.2159 (8)	0.3851 (4)	0.5490 (4)	0.0494 (16)	
H14A	0.2020	0.3223	0.5681	0.059*	
C15	0.2011 (7)	0.4721 (4)	0.5916 (3)	0.0375 (12)	
C16	0.1738 (8)	0.4681 (4)	0.6613 (3)	0.0463 (15)	
H16A	0.1622	0.4071	0.6832	0.056*	
C17	0.1642 (9)	0.5552 (5)	0.6972 (3)	0.0507 (15)	
H17A	0.1518	0.5538	0.7441	0.061*	
C18	0.1734 (8)	0.6465 (4)	0.6628 (3)	0.0422 (13)	
H18A	0.1594	0.7050	0.6875	0.051*	
C19	0.2191 (7)	0.5668 (4)	0.5611 (3)	0.0317 (11)	
C20	0.2586 (7)	0.5750 (4)	0.4907 (3)	0.0324 (11)	
N3	0.2010 (6)	0.6526 (3)	0.5969 (2)	0.0324 (9)	
O7	0.4073 (5)	0.8742 (2)	0.47182 (17)	0.0345 (8)	
H7A	0.3512	0.9298	0.4780	0.041*	
H7B	0.5130	0.8761	0.4958	0.041*	

### Atomic displacement parameters (Å^2^)

**Table:**

	*U*^11^	*U*^22^	*U*^33^	*U*^12^	*U*^13^	*U*^23^
Ni1	0.0348 (3)	0.0194 (3)	0.0314 (3)	0.0007 (3)	−0.0033 (2)	−0.0022 (3)
N1	0.034 (2)	0.0173 (18)	0.028 (2)	0.0010 (16)	−0.0053 (17)	−0.0006 (15)
C1	0.040 (3)	0.020 (2)	0.033 (3)	0.001 (2)	−0.005 (2)	0.0031 (19)
C2	0.034 (3)	0.023 (2)	0.034 (3)	0.000 (2)	−0.008 (2)	−0.001 (2)
C3	0.036 (3)	0.021 (2)	0.028 (2)	−0.002 (2)	−0.002 (2)	−0.0005 (19)
C4	0.038 (3)	0.022 (2)	0.028 (2)	0.002 (2)	−0.005 (2)	0.0019 (19)
C5	0.030 (2)	0.020 (2)	0.028 (2)	0.0004 (18)	−0.0018 (19)	0.0029 (18)
C6	0.037 (3)	0.019 (2)	0.031 (3)	−0.0007 (19)	−0.007 (2)	0.0048 (19)
O1	0.0378 (19)	0.0238 (17)	0.040 (2)	0.0042 (15)	−0.0057 (15)	−0.0025 (15)
O2	0.049 (2)	0.0286 (19)	0.046 (2)	0.0032 (17)	−0.0203 (18)	0.0017 (16)
C7	0.047 (3)	0.025 (2)	0.030 (3)	−0.002 (2)	−0.005 (2)	−0.003 (2)
O3	0.068 (3)	0.039 (2)	0.069 (3)	0.011 (2)	−0.035 (2)	−0.028 (2)
O4	0.057 (3)	0.031 (2)	0.059 (3)	0.0141 (19)	−0.021 (2)	−0.0126 (19)
C8	0.036 (3)	0.021 (2)	0.025 (2)	−0.003 (2)	−0.003 (2)	0.0037 (18)
O5	0.0275 (18)	0.0263 (18)	0.047 (2)	0.0016 (15)	−0.0073 (15)	−0.0007 (15)
O6	0.038 (2)	0.0237 (17)	0.039 (2)	0.0017 (15)	−0.0099 (15)	−0.0071 (15)
N2	0.033 (2)	0.028 (2)	0.036 (2)	0.0024 (17)	−0.0039 (18)	−0.0058 (18)
C9	0.043 (3)	0.042 (3)	0.041 (3)	0.003 (3)	−0.001 (2)	−0.008 (3)
C10	0.052 (4)	0.058 (4)	0.038 (3)	0.008 (3)	−0.009 (3)	−0.014 (3)
C11	0.043 (3)	0.046 (4)	0.060 (4)	0.008 (3)	−0.008 (3)	−0.029 (3)
C12	0.028 (3)	0.033 (3)	0.060 (4)	0.005 (2)	−0.011 (2)	−0.015 (3)
C13	0.034 (3)	0.028 (3)	0.083 (5)	0.005 (2)	−0.011 (3)	−0.015 (3)
C14	0.038 (3)	0.020 (3)	0.090 (5)	0.000 (2)	−0.004 (3)	0.000 (3)
C15	0.026 (3)	0.030 (3)	0.057 (3)	0.001 (2)	−0.003 (2)	0.006 (2)
C16	0.033 (3)	0.035 (3)	0.070 (4)	−0.001 (2)	0.004 (3)	0.019 (3)
C17	0.050 (4)	0.052 (4)	0.050 (4)	0.001 (3)	0.005 (3)	0.018 (3)
C18	0.048 (3)	0.040 (3)	0.039 (3)	0.001 (3)	0.007 (2)	0.001 (2)
C19	0.026 (2)	0.025 (2)	0.044 (3)	−0.0004 (19)	−0.008 (2)	−0.003 (2)
C20	0.029 (3)	0.024 (2)	0.043 (3)	0.003 (2)	−0.010 (2)	−0.006 (2)
N3	0.040 (2)	0.024 (2)	0.034 (2)	−0.0017 (18)	−0.0001 (18)	0.0019 (17)
O7	0.039 (2)	0.0264 (18)	0.0378 (19)	0.0009 (15)	−0.0046 (15)	−0.0006 (15)

### Geometric parameters (Å, °)

**Table:**

Ni1—N1	2.001 (4)	N2—C20	1.349 (6)
Ni1—N2	2.053 (4)	C9—C10	1.403 (8)
Ni1—N3	2.081 (4)	C9—H9A	0.9300
Ni1—O7	2.108 (4)	C10—C11	1.355 (9)
Ni1—O6	2.115 (3)	C10—H10A	0.9300
Ni1—O1	2.184 (3)	C11—C12	1.395 (8)
N1—C5	1.329 (6)	C11—H11A	0.9300
N1—C1	1.331 (6)	C12—C20	1.402 (7)
C1—C2	1.381 (6)	C12—C13	1.435 (8)
C1—C6	1.522 (7)	C13—C14	1.352 (9)
C2—C3	1.393 (7)	C13—H13A	0.9300
C2—H2A	0.9300	C14—C15	1.442 (8)
C3—C4	1.398 (6)	C14—H14A	0.9300
C3—C7	1.510 (7)	C15—C16	1.391 (8)
C4—C5	1.380 (6)	C15—C19	1.413 (7)
C4—H4A	0.9300	C16—C17	1.368 (9)
C5—C8	1.522 (6)	C16—H16A	0.9300
C6—O1	1.254 (6)	C17—C18	1.401 (8)
C6—O2	1.258 (6)	C17—H17A	0.9300
C7—O4	1.203 (6)	C18—N3	1.320 (6)
C7—O3	1.302 (6)	C18—H18A	0.9300
O3—H3A	0.8512	C19—N3	1.358 (6)
C8—O5	1.253 (6)	C19—C20	1.425 (7)
C8—O6	1.261 (6)	O7—H7A	0.8502
N2—C9	1.325 (7)	O7—H7B	0.8498
			
N1—Ni1—N2	172.90 (16)	C9—N2—C20	118.0 (4)
N1—Ni1—N3	103.18 (16)	C9—N2—Ni1	128.3 (4)
N2—Ni1—N3	80.03 (17)	C20—N2—Ni1	113.7 (3)
N1—Ni1—O7	90.09 (15)	N2—C9—C10	122.0 (6)
N2—Ni1—O7	88.24 (15)	N2—C9—H9A	119.0
N3—Ni1—O7	161.85 (15)	C10—C9—H9A	119.0
N1—Ni1—O6	77.69 (14)	C11—C10—C9	119.8 (6)
N2—Ni1—O6	95.55 (14)	C11—C10—H10A	120.1
N3—Ni1—O6	100.60 (16)	C9—C10—H10A	120.1
O7—Ni1—O6	94.23 (14)	C10—C11—C12	119.7 (5)
N1—Ni1—O1	76.68 (14)	C10—C11—H11A	120.2
N2—Ni1—O1	110.16 (15)	C12—C11—H11A	120.2
N3—Ni1—O1	82.81 (15)	C11—C12—C20	117.0 (5)
O7—Ni1—O1	88.39 (13)	C11—C12—C13	123.8 (5)
O6—Ni1—O1	154.24 (13)	C20—C12—C13	119.2 (5)
C5—N1—C1	121.8 (4)	C14—C13—C12	120.8 (5)
C5—N1—Ni1	118.1 (3)	C14—C13—H13A	119.6
C1—N1—Ni1	119.1 (3)	C12—C13—H13A	119.6
N1—C1—C2	120.8 (4)	C13—C14—C15	121.4 (5)
N1—C1—C6	112.8 (4)	C13—C14—H14A	119.3
C2—C1—C6	126.4 (4)	C15—C14—H14A	119.3
C1—C2—C3	118.3 (4)	C16—C15—C19	118.0 (5)
C1—C2—H2A	120.8	C16—C15—C14	123.8 (5)
C3—C2—H2A	120.8	C19—C15—C14	118.2 (5)
C2—C3—C4	119.9 (4)	C17—C16—C15	119.1 (5)
C2—C3—C7	121.6 (4)	C17—C16—H16A	120.5
C4—C3—C7	118.5 (4)	C15—C16—H16A	120.5
C5—C4—C3	118.0 (4)	C16—C17—C18	119.6 (6)
C5—C4—H4A	121.0	C16—C17—H17A	120.2
C3—C4—H4A	121.0	C18—C17—H17A	120.2
N1—C5—C4	121.2 (4)	N3—C18—C17	122.6 (5)
N1—C5—C8	112.7 (4)	N3—C18—H18A	118.7
C4—C5—C8	126.0 (4)	C17—C18—H18A	118.7
O1—C6—O2	126.7 (5)	N3—C19—C15	122.2 (5)
O1—C6—C1	116.1 (4)	N3—C19—C20	117.5 (4)
O2—C6—C1	117.1 (4)	C15—C19—C20	120.3 (5)
C6—O1—Ni1	114.3 (3)	N2—C20—C12	123.4 (5)
O4—C7—O3	125.1 (5)	N2—C20—C19	116.6 (4)
O4—C7—C3	122.3 (5)	C12—C20—C19	120.0 (5)
O3—C7—C3	112.6 (4)	C18—N3—C19	118.4 (5)
C7—O3—H3A	120.5	C18—N3—Ni1	129.1 (4)
O5—C8—O6	126.0 (4)	C19—N3—Ni1	111.8 (3)
O5—C8—C5	118.3 (4)	Ni1—O7—H7A	103.6
O6—C8—C5	115.7 (4)	Ni1—O7—H7B	94.0
C8—O6—Ni1	115.4 (3)	H7A—O7—H7B	105.9

### Hydrogen-bond geometry (Å, °)

**Table:**

*D*—H···*A*	*D*—H	H···*A*	*D*···*A*	*D*—H···*A*
O3—H3A···O2^i^	0.85	1.70	2.550 (5)	178
O7—H7A···O5^ii^	0.85	1.87	2.702 (5)	167
O7—H7B···O5^iii^	0.85	2.00	2.783 (5)	152

Symmetry codes: (i) −*x*+1, *y*+1/2, −*z*+3/2; (ii) −*x*, −*y*+2, −*z*+1; (iii) *x*+1, *y*, *z*.
